# Testing the TEC-MED-Integrated Transcultural Social–Ethical-Care Model for Older People in the Mediterranean Basin: A Mixed-Method Quasiexperimental Study Protocol

**DOI:** 10.5334/ijic.9017

**Published:** 2025-05-09

**Authors:** Marta Lima-Serrano, Regina Allande-Cussó, Ana Magdalena Vargas-Martínez, Georges Karam, Lea El-Korh, Ana María Porcel-Gálvez

**Affiliations:** 1 Department of Nursing, Faculty of Nursing, Physiotherapy, and Podiatry, University of Seville, 9 Avenzoar, 41009, Seville, Spain; 2 Institute for Development, Research, Advocacy and Applied Care, St. George Hospital Street, Beirut, Lebanon

**Keywords:** TEC-MED Project, longitudinal analytical design, quasiexperimental design, economic evaluation, mixed-method approach

## Abstract

**Background:**

The global shift toward aging populations, driven by advancements in the economy, healthcare, and society, has transformed once-lethal diseases into chronic conditions. Complex patient management scenarios emerge through this trend, coupled with demographic changes, given that multiple chronic diseases coexist within an individual. The TEC-MED project, which spans multiple countries, aims to evaluate whether the TEC-MED model can improve the quality of life and reduce care dependency among older adults in the Mediterranean region.

**Methods:**

This study employs a mixed-method approach including a longitudinal analytical quasiexperimental design, economic evaluation, and qualitative techniques such as interviews and focus groups. The sample includes at least 20,000 older adults and their caregivers from the Mediterranean basin countries. Quantitative data analysis encompasses descriptive and bivariate statistical analyses, regression models, and economic evaluation, whereas qualitative analysis involves discourse analysis and thematic categorization.

**Discussion:**

The TEC-MED project’s focus on integrating social and health care is expected to improve health outcomes and quality of life for older people and their caregivers. The anticipated outcomes will contribute valuable insights into the TEC-MED model’s effectiveness in promoting person-centered care and addressing global challenges posed by aging populations.

**Trial registration:**

NCT06184178 (ClinicalTrials.gov).

**Date of registration:**

27/12/2023.

## Introduction

The global trend of aging populations, coupled with demographic shifts in developed nations resulting from advancements in economy, healthcare, and society, has led to the transformation of once-lethal diseases, such as ischemic heart disease, into chronic conditions. Consequently, managing patients has become more complex as new complications and interactions among multiple chronic diseases have emerged within an individual [1]. Globally, multimorbidity (with two or more chronic diseases) affects over 60% of the older population [2], with implications for disability, depression, hospitalizations, and mortality [3,4,5,6]. In a cross-sectional population-based study conducted using data from wave 6 release guide 7.0.0 of the Survey of Health, Aging, and Retirement in Europe, the prevalence of multimorbidity among 21,458 individuals across 17 European countries was approximately 33%; this study is the first multidisciplinary, cross-country, longitudinal research project in Europe. In line with these findings, people with high levels of loneliness and low quality of life have a higher multimorbidity prevalence. Additionally, survival is reduced in people with low social support [7]. The increasing prevalence of chronic diseases is closely linked to age, underscoring the importance of promoting self-care across the lifespan [8,9,10,11]. However, the self-care capacity of individuals is hindered not only by multimorbidity with the consequent greater vulnerability, the emergence of pathological processes, and the subsequent decrease in functionality [12,13,14] but also by loneliness and other factors that influence an increase in the risk of social exclusion in this population. The ongoing issue of social exclusion poses a significant challenge for aging societies in Europe and other regions [15]. Addressing this issue requires adjustments at both the healthcare sector and societal levels [12,13,14]. The gap between the assistance provided to dependent individuals and their capacity for self-care needs to be bridged [16]. Many older adults and their families resort to long-term care facilities to ensure that essential care is provided [16]. Long-term care must focus on maximizing individuals’ capabilities across their lifespan, providing a supportive setting, and providing essential care to sustain and enhance functional abilities [13]. Considering their requirements, values, and expectations, this type of care should recognize these individuals as distinct entities, delivering person-centered care [11,13].

Within this framework, care models that address the unique requirements of specific populations should be developed and implemented to enhance their quality of life, independence, and citizen empowerment for self-care [17]. These models should also consider the physical, cognitive, emotional, and social aspects [17].

A recent review of person-centered care in long-term settings highlights its benefits, including enhanced quality of life, well-being, and reduced neuropsychiatric symptoms. However, methodological disparities lead to inconsistent findings [18]. Robust cross-cultural studies with standardized scales are vital for identifying at-risk groups and empowering the chronically ill. To foster cross-country understanding, policies must be aligned with citizens’ needs by analyzing health outcomes by care level and involving diverse stakeholders [18].

Optimizing health outcomes for patients needing complex care requires the vertical and horizontal integration of services, bridging the healthcare and social sectors [19]. Failure to connect with necessary services results in adverse experiences, health deterioration, and costly interventions [20,21,22].

Exemplary programs in Canada prioritize integrating services for older adults, yet achieving comprehensive integration across health, social, and community sectors remains challenging [23]. Current integration efforts are confined to traditional health services, and national policies must expand to include social services for comprehensive primary healthcare [23]. Intentional connections between healthcare and social services are crucial to achieving comprehensive primary care for community-dwelling older adults [24].

Increased interprofessional collaboration addresses healthcare challenges caused by aging populations. Interprofessional collaborative practice involving diverse health professionals improves health service outcomes. According to the World Health Organization (WHO), collaborative practice has a positive impact on patient satisfaction and health outcomes [25]. In Vietnam, where significant healthcare demands exist, collaborative approaches are promising. Vo et al. (2023) identified challenges in geriatric care and highlighted collaborative practices as essential solutions. Collaboration is crucial in addressing healthcare system struggles, engaging stakeholders, and clarifying roles. Thus, the effectiveness of collaboration between healthcare and social care professionals in promoting older adults’ health and well-being should be explored [26].

**Figure 1:**
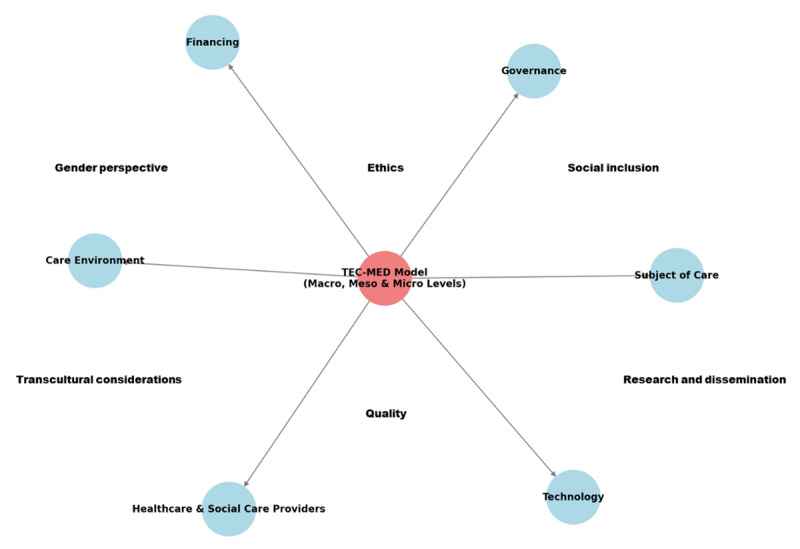
Model’s theoretical framework.

Given the lack of interventions integrating healthcare and social care services for older adults and the shared trends and care needs of these individuals in Mediterranean countries, the TEC-MED project was developed, encompassing Egypt, Greece, Lebanon, Spain, and Tunisia. This project aims to pilot transcultural social–ethical care under the TEC-MED care model [27], which integrates social and health care for older adults and their caregivers, and to assess its impact on improving the quality of life and other health outcomes. The outline of the theoretical framework of the starting model (Figure 1), which will be refined and developed during the implementation of the project, focuses on care for older individuals who are 60 years and over, dependent, and at risk of social exclusion. Unlike previous models such as Leininger’s Transcultural Nursing [28], Pender’s Health Promotion [29], or Person-centered Care Framework [30], the TEC-MED model incorporates a holistic and integrated perspective, addressing gaps in considering the caregivers’ and families’ perspectives, ethics, technology, and financing. It includes dimensions such as the subject of care, healthcare and social care providers, care environment, governance, financing, and technology. Each dimension operates at the macro, meso, and micro levels. Additionally, the model is characterized by five key elements: quality, research, and dissemination; gender perspective; ethics; social inclusion; and transcultural considerations. These elements influence the conceptualization of the model and contribute to its distinctiveness [27].

## Methods

### Study design

This study employs a mixed-method approach including a longitudinal analytical quasiexperimental design, economic evaluation, and qualitative techniques such as interviews and focus groups.

Regarding the quasiexperimental design, an intervention group (IG) will be established from a sample size of at least 20,000 subjects from all participating countries, and a minimum of 25% of this population will comprise the control group (CG), who will not receive the intervention initially but will do so at the end of the trial to safeguard ethical principles in research.

**Figure 2:**
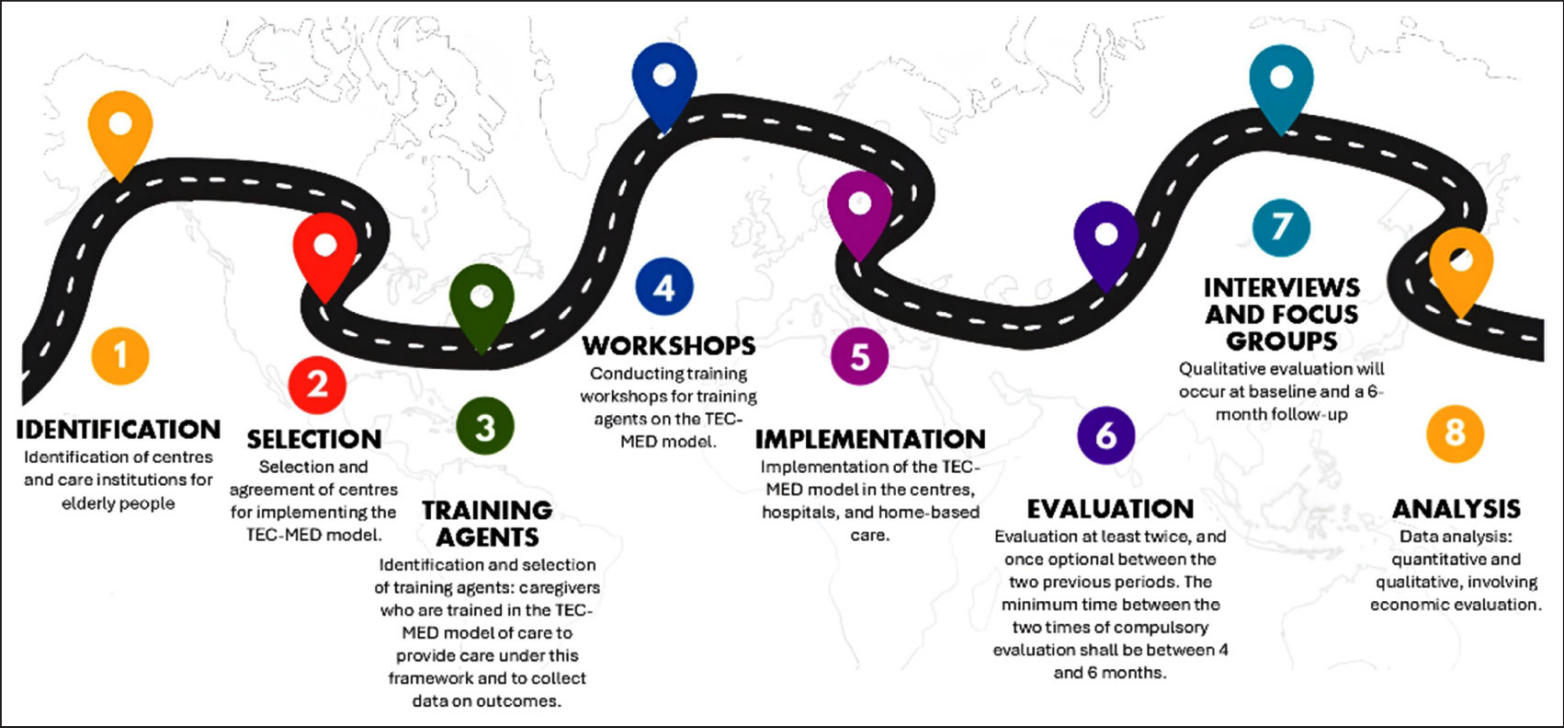
Stages of the project.

**Figure 3:**
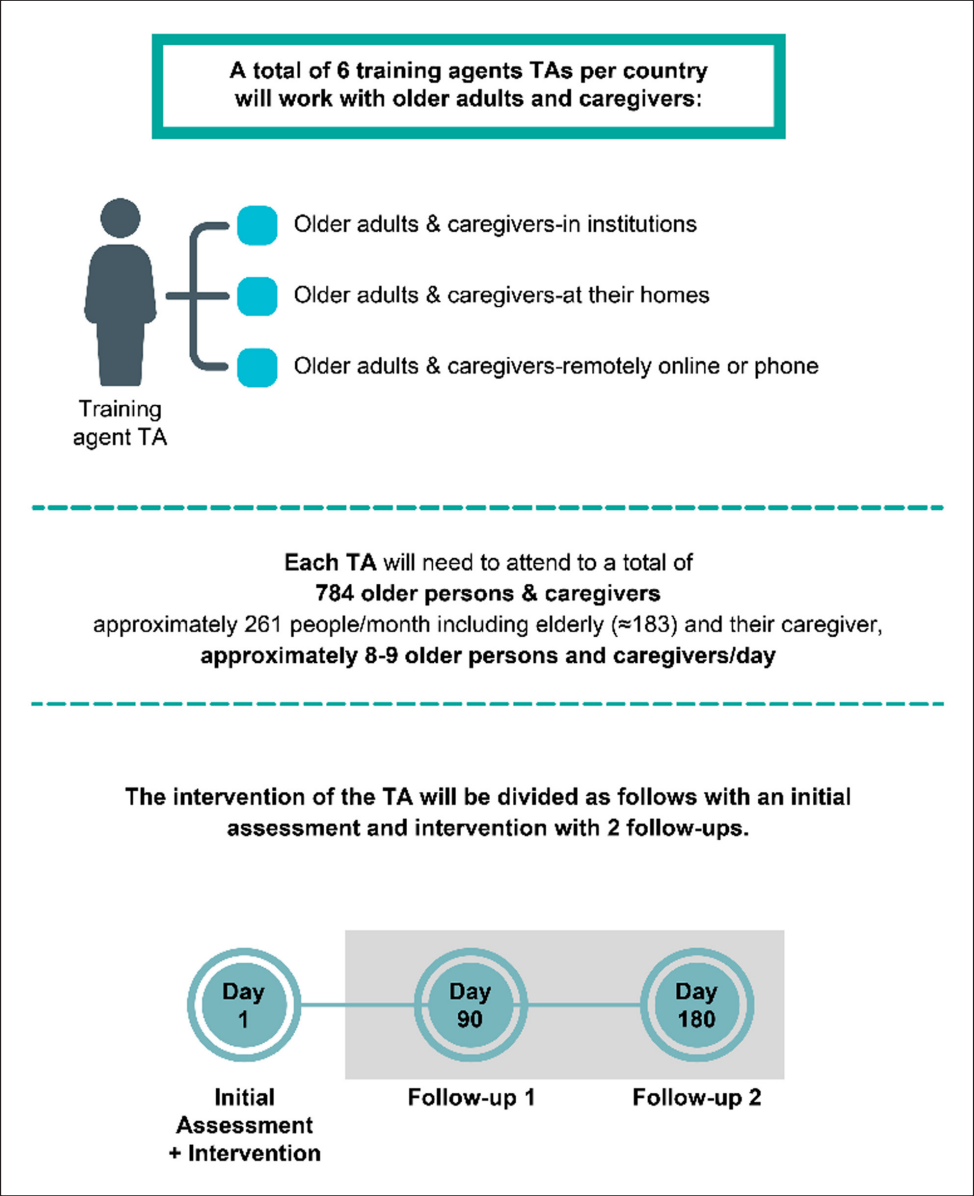
Expected work of the training agents.

The study will be conducted over 3 years in several stages (Figure 2):

1. Identification of centers and care institutions for older adults.
2. Selection and agreement of centers for TEC-MED model implementation.
3. Identification and selection of training agents (caregivers trained on the TEC-MED model to provide care under this framework and collect data on outcomes).
4. Training workshops for training agents on the TEC-MED model. Each participating country will organize at least four training sessions, with a total duration of 40 hours (Figure 3). The training sessions, central to the TEC-MED model’s implementation, will include comprehensive content designed to standardize care practices across participating countries. Each session will cover the theoretical framework of the TEC-MED model, the practical use of tools for data collection, and specific skills to identify and address risk factors such as social exclusion and dependency levels. Session content will include the project’s objectives and presentation, the evaluation variables, and the concrete procedure of data collection. Evaluation of the training sessions will involve pre-and post-assessments to measure improvements in caregivers’ knowledge, skills, and confidence. Additionally, a follow-up evaluation will assess the long-term retention of skills and the practical application of the TEC-MED model during the intervention phase. Standardized checklists and self-reported surveys will ensure consistency and comparability of training outcomes across all countries.
5. Implementation of the TEC-MED model in centers, hospitals, and home-based care.
6. Evaluation at least twice and once optional between the two previous periods. The minimum time between the two compulsory evaluations shall be between 4 and 6 months. The optional assessment, if any, shall be after 3 months from the baseline measurement.
7. Qualitative assessment: interviews and focus groups. Qualitative evaluation will occur at baseline and 6 months after for follow-up to analyze the perception of the pilot study participants about the implementation process and the impact of the TEC-MED model, identifying areas to improve. The interviews will be conducted in the local language, using a script validated by all partners. Each partner country will implement four focus groups, one for each profile: final beneficiary, caregiver, professional caregiver, and stakeholder. Both the interviews and the focus groups will be audio-recorded for transcription categorization and analysis. The focus groups will be transcribed and translated into English to redact a summary of the main points of the discussion; the summary will be shared with the partners afterward.
8. Data analysis: quantitative and qualitative, involving economic evaluation. Qualitative and quantitative data integration will follow a triangulation design to provide a comprehensive understanding of the TEC-MED model’s impact. Quantitative data will be analyzed to identify statistical trends and correlations between intervention activities and health outcomes. Concurrently, qualitative data, collected through interviews and focus groups, will provide contextual insights into participants’ perceptions of the care model. These two datasets will be combined during the discussion phase to cross-validate findings and enhance the depth of interpretation. In this sense, qualitative themes such as participant satisfaction with the intervention will be juxtaposed with quantitative outcomes like quality-of-life improvements. A joint display matrix will be utilized to visually represent how qualitative and quantitative data converge, diverge, or complement one another, ensuring a cohesive and nuanced analysis of the TEC-MED model’s effectiveness.

### Intervention

The quality of the implementation of the model will be assessed using the Donabedian model [31], which describes the quality of care according to three categories:

1. Structure (the context in which care is delivered, including staff, financing, and equipment)
2. Process (the transactions between patients and providers throughout the delivery of healthcare)
3. Outcomes (the healthcare effects on the health status of patients and populations)

**Figure 4:**
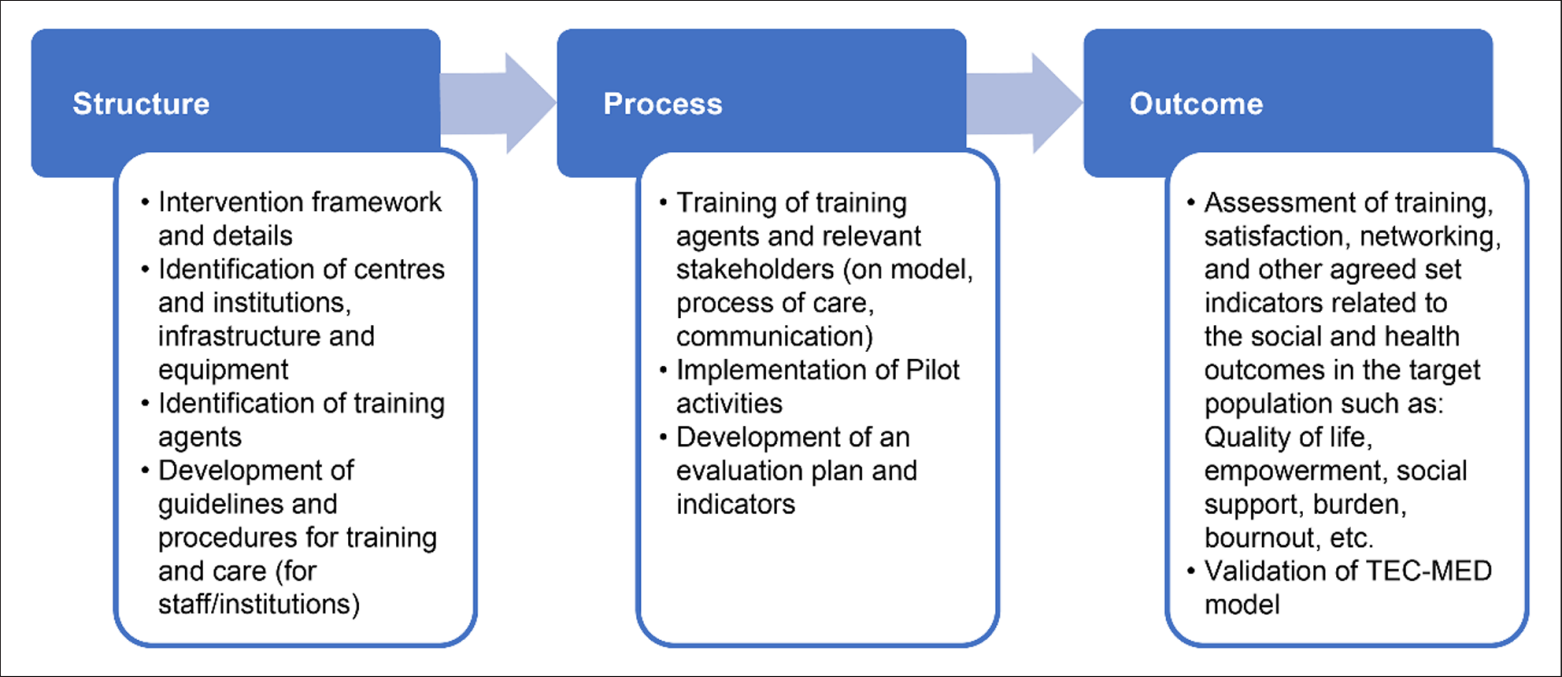
Activities of the project.

Based on the Donabedian model for the TEC-MED model implementation, the following activities should be implemented (Figure 4).

The intervention is expected to have two main components: assessment and intervention. The assessment component will include screenings using the agreed scales integrated into the platform, and the intervention component will be based on the assessment results; therefore, if any of the screenings are positive, an intervention will occur, and recommendations will be given to address the issues.

**Figure 5:**
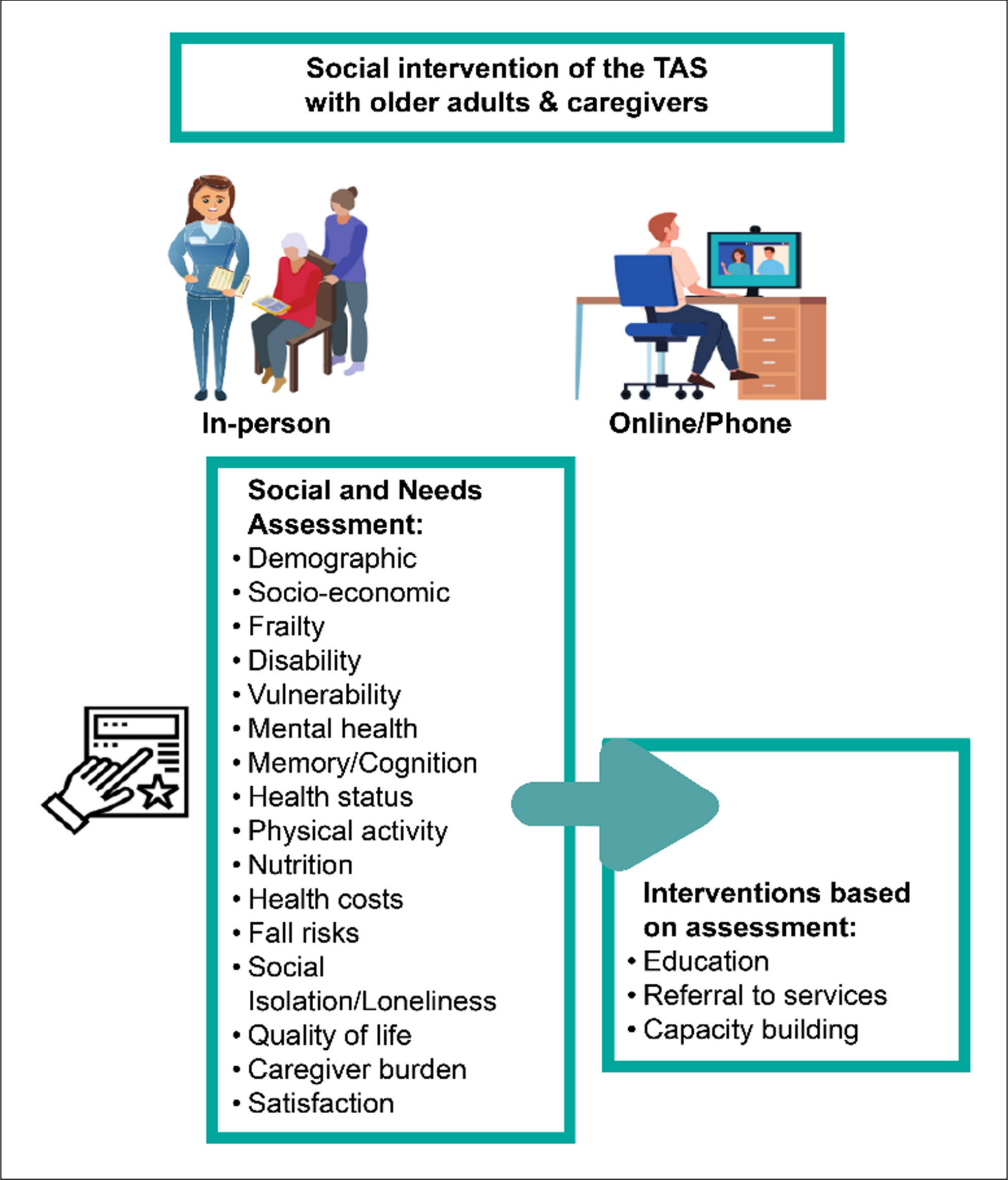
The intervention overview.

Specific interventions will be selected based on the Nursing Intervention Classification [32] and the expected outcomes will be based on the Nursing Outcomes Classification [33]. Likewise, the NANDA-International Taxonomy of Nursing Diagnoses [34] will be used to identify the problems that will form the basis for the intervention design. Interventions will be in the form of education, referral to services, contact with relevant institutions, and capacity building, among others (Figure 5). When modifying the intervention, each country should keep in mind the agreed-upon general framework and the goal of decreasing social isolation and exclusion.

### Population and sample

The population will consist of older adults who are over 60 years old, dependent, and at risk of needing social care, and their caregivers from Spain, Italy, Greece, Lebanon, Egypt, and Tunisia. The sample will be selected with an approximate ratio of 70% in the IG vs. 30% in the CG, using consecutive convenience sampling. Likewise, 60% of the sample will be subjects residing at home and 40% in nursing homes.

Regarding the quantitative assessment and the sample of older people, the inclusion criteria will be based on age (must be 60 years or older [32]), the level of dependency in care (low or moderate, assessed by the Inventory of the Care Dependency level using Nursing Outcomes-INICIARE scale) [33,34,35,36], the risk of social exclusion (assessed by the At Risk or Poverty -AROPE index), and location [37]. Participants may also include individuals receiving home-based care, those residing in nursing homes, or those hospitalized. Conversely, the exclusion criteria encompass severe dependency, palliative care needs, deafness, severe cognitive impairment, specific medical conditions, and medications affecting the cognitive status. As for the sample of caregivers, all those caregivers of the older person who fulfill the inclusion criteria and agree to participate will be included, without any exclusion criteria related to age.

For the qualitative methodology, the following profiles represented in the TEC-MED model will be considered: institutional and nongovernmental organization (NGO) staff, nonprofessional caregivers, final beneficiaries, community association leaders, and training agents. The latter were professionals providing care to older adults who received specific training to implement care under the new TEC-MED model. The saturation criteria will be considered for the sample size. However, at least 16 interviews will be conducted in each participating country, considering the adequate representation of men and women. Focus groups will be implemented, involving six to eight participants. Participants and key informants will be identified through theoretical saturation sampling among stakeholders around care provision, management, and education, as well as older adults and caregivers participating in the project. Institutional and NGO staff, nonprofessional caregivers, and final beneficiaries who will participate in the IG, training agents, and other stakeholders who will be introduced to the TEC-MED model will be selected.

### Variables

The study variables are divided into sociodemographic, scale variables, and economic evaluation. Regarding the sociodemographic variables, older adults’ and caregivers’ data on sex, age, country of origin, country of residence, and ethnicity will be collected.

For the scale variables, older adults’ score data from the following tools will be collected: the Inventory of the Care Dependency level using the Nursing Outcomes-INICIARE scale, At Risk or Poverty – AROPE index, Quality of Life -QoL (EQ-5D-5L) scale, Functional Social Support Questionnaire- DUKE-UNC, HLS-EU-Q16 questionnaire, and Self-perception of family health- AESFA scale. The INICIARE scale identifies the levels of care dependency using a Likert scale from 1 to 5 points and has high reliability (Cronbach’s alpha: 0.91) and validity (74% of the explanatory power of variance) [35,36]. The INICIARE scale, previously validated in a similar demographic, will be re-evaluated for its applicability in diverse Mediterranean settings to ensure reliability. The AROPE index is the main indicator to monitor the EU 2030 target on poverty and social exclusion and is divided into three sub-indicators: 1) low work intensity, having worked <20% of the hours available (for their members in working age); 2) at risk of poverty, earning <60% of the national median income per consumption unit; 3) risk of material deprivation (MD), lacking three or more of the necessary items from a list of nine [37]. The QoL (EQ-5D-5L) scale provides a simple descriptive profile and a single index value for health status; it consists of a weighted sum of five dimensions: mobility, self-care, usual activities, pain/discomfort, and anxiety/depression [38]. The DUKE-UNC Functional Social Support Questionnaire is an 11-item questionnaire that assesses the level of perceived social support, with reliability coefficients of 0.80 and 0.92 for each factor [39]. Furthermore, the HLS-EU-Q16 questionnaire consists of 16 questions that classify the degree of difficulty perceived by the respondent in each task or situation about health literacy, and it has a Cronbach’s alpha value of 0.98 [40]. Lastly, the AESFA scale, which has 24 items divided into five factors, measures the self-perception of family health status, with a Cronbach’s alpha of 0.87 [41].

In the sample of caregivers, the Caregiver Strain Index of 13 items will be added. It examines both subjective and objective elements of caregiver strain, with good internal reliability (alpha coefficient: 0.90) [42].

Each scale in the language of each participating country was validated, and permissions were obtained from the authors for their use in this project.

The variables used for the economic evaluation related to resource utilization will be collected using a questionnaire aimed at caregivers and final TEC-MED care model users (older people) at baseline and the 6-month follow-up period. This questionnaire was designed from questions taken from the Resource Utilization in Dementia instrument [43] as well as from the National Health Survey in Spain [44] and European Health Survey [45] for questions related to the current job situation and profession.

This study assumes that the selected instruments are culturally adaptable across Mediterranean populations, which may not fully account for regional variations.

### Data collection

Trained training agents will collect the data once the participant and the caregiver provide informed consent. After agreement with the participating centers and associations in each country, they will visit the homes of older adults and conduct an initial assessment based on the study variables. This data will be collected on a web platform, which will be specifically designed, to guarantee data confidentiality and security. Likewise, they will assess each participant’s caregiver (Figure 2).

Once this initial assessment has been conducted, they will implement socio-health care based on the TEC-MED care model through health education sessions, risk situation identification, referral, if appropriate, and referral to participation in community support resources, if possible.

**Figure 6:**
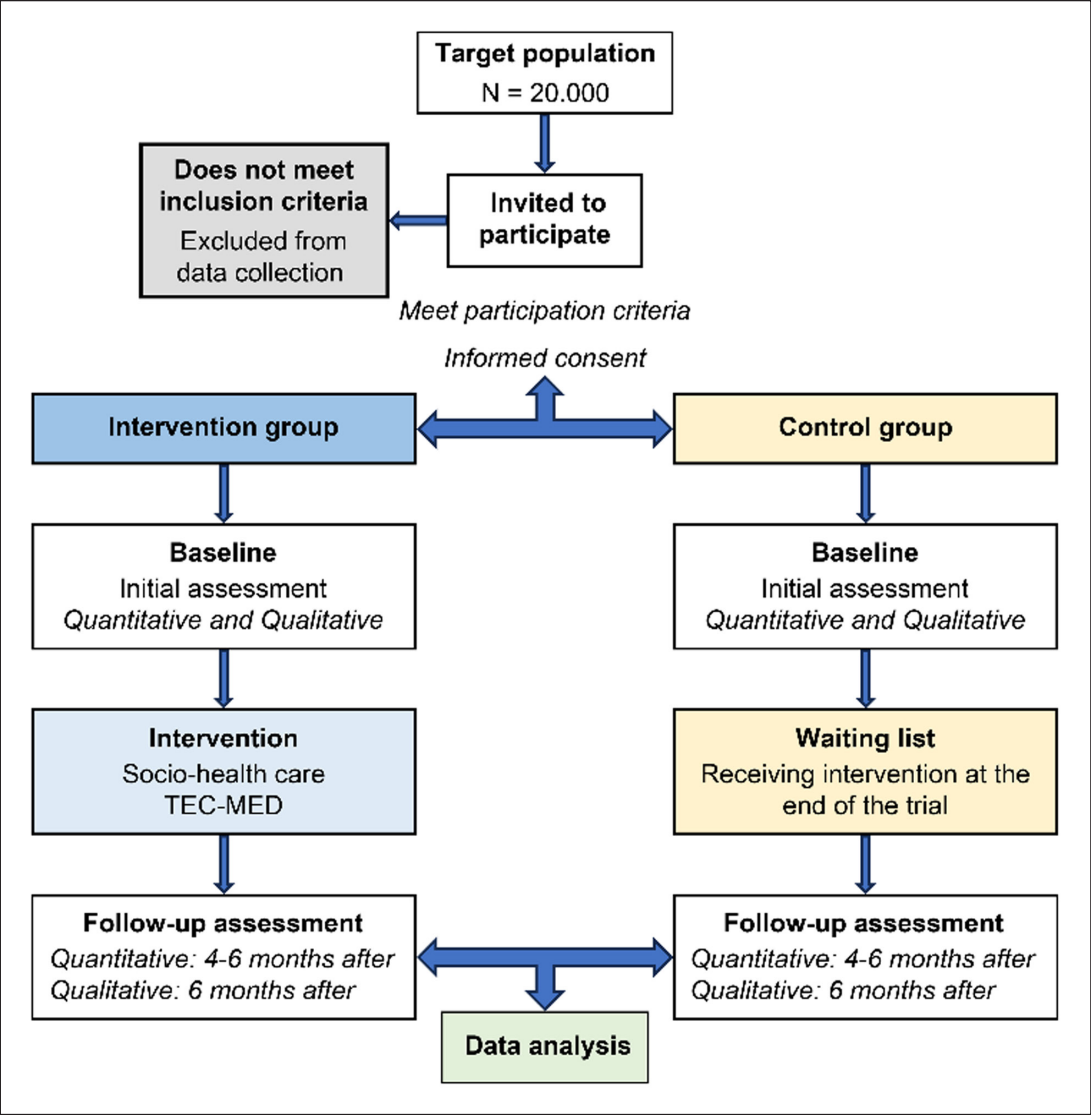
Data collection diagram.

The same training agents will perform at least a second assessment 4–6 months after the first assessment, both for the participants and the caregiver, consistently recording data on the web platform created for this purpose (Figure 6).

### Data analysis

Collected data will undergo descriptive and bivariate statistical analysis and tests for data normality. Statistical significance will be set at p < 0.05 with a 95% confidence interval. Regression studies will be conducted, considering the study’s longitudinal nature. Quantitative data will be analyzed using the SPSS V29 software. The necessary data imputation techniques should be performed on the collected database.

Regarding the economic evaluation, the cost-effectiveness and cost-utility of the TEC-MED model will be assessed using the incremental cost-effectiveness ratio as a final measure. The health outcome measure taken for the cost-effectiveness analysis will be the reduction or maintenance of the care dependency level and the reduction of hospital admissions, falls, and social exclusion. For the cost-utility analysis, it will be the quality-adjusted life years calculated from survival and the utility indices of the generic questionnaire that measures the quality of life using the EuroQoL scale. The uncertainty will be measured through deterministic and probabilistic sensitivity analyses.

Content analysis will be conducted to generate the most prevalent thematic categories for qualitative data obtained through semistructured interviews and focus groups. Additionally, AtlasTi© or NUDIST© are examples of software that can be used to support qualitative analysis.

### Ethical considerations

The study has received the ethical approval of the Research Ethics Committee of the Junta de Andalucía in Spain, given that the coordinating organization of the consortium is the University of Seville in Spain (Reference no.: 2412-N-19) for its execution. Ethical approval was also obtained from the ethics reference committees of Tunisia (Reference no.: 01/2022), Egypt (Reference no.: HU.REC.H.6-22), Lebanon (Reference no.: IRB-REC/Ol05l-2112321), Greece (Reference no.: 40640/30-5-22), and Italy (Reference no.: 18/03/22). Furthermore, this study adheres to the guidelines outlined in the Declaration of Helsinki for studies involving human subjects, as ratified in 2013 [46].

Informed consent will be obtained from all study participants, emphasizing the voluntary nature of participation and data anonymity. Given the study involves older adults, special considerations were taken to ensure comprehension and voluntary participation, including the use of simplified consent forms reviewed by ethical committees.

A protocol for the custody and treatment of personal data was also developed. This protocol was approved by the Data Protection Department of the University of Seville, Spain, the lead country in this project. Therefore, data collected in the context of this study will remain under the custody of the research team and will not be shared with third parties.

## Discussion

This project aims to evaluate the impact of care provision under the TEC-MED model on health outcomes such as quality of life, care dependency level, health literacy, and caregiver strain. These data should allow to investigate the TEC-MED model’s effectiveness [27] while providing evidence about the improvement of integrated care for older people and their caregivers in the Mediterranean region. In this sense, evidence indicates that integrated care models need to be studied in specific contexts to understand their effectiveness under a specific demographic and cultural situation [47]. Additionally, care delivery that integrates health and social care, such as the TEC-MED model, could prevent adverse events by improving communication between health and social care providers [48]. Likewise, the proposal of a mixed-method research design is a strength of the project because it facilitates a more comprehensive approach to impact [49].

The findings of the TEC-MED study should be contextualized within the existing literature on integrated care models for older adults. Previous research has demonstrated that integrating health and social care services enhances quality of life and reduces caregiver burden [50,51]. For instance, models implemented in Canada have shown significant improvements in service coordination, although structural barriers to full integration persist [51]. In this regard, the TEC-MED model not only incorporates the well-documented principles of integration but also introduces a distinctive transcultural and ethical approach. Unlike previous models such as that developed by Valentijn et al [52], which focused on primary care, the TEC-MED model specifically addresses the Mediterranean context, considering factors such as social exclusion and gender perspectives in care provision.

Moreover, the literature has highlighted the need for interventions that not only integrate services but also respond to the sociocultural specificities of the target populations [53,54]. While Vo et al. (2023) emphasize the importance of interdisciplinary collaboration in geriatric care in Vietnam [54], the TEC-MED model translates this principle into a transcultural approach encompassing diverse Mediterranean countries with heterogeneous healthcare systems. This broader framework enhances the model’s adaptability to different settings, facilitating the implementation of context-specific strategies that promote equity in the care of older adults at risk of social exclusion.

However, the study faces significant challenges related to external variables, such as changes in healthcare policies, economic fluctuations, and unforeseen events, which could impact the results and complicate the attribution of specific modifications to the TEC-MED model. Additionally, incorporating economic evaluation has inherent limitations, given that the results are subject to uncertainty because of assumptions and variability in costs and benefits, potentially affecting the generalization of findings to different contexts. Another limitation includes potential selection bias due to the non-randomized design of the control group. Likewise, despite the emphasis on interprofessional collaboration, the study acknowledges that effective coordination among healthcare and social services professionals may be difficult to achieve, with barriers in communication and ambiguous roles that could impact the successful implementation of the TEC-MED model.

## Conclusions

This project seeks to provide evidence regarding implementing and evaluating the TEC-MED integrated care model for older individuals and their caregivers in Mediterranean basin countries. The outcomes are expected to help identify areas for improvement in the socio-health care system for older adults and their caregivers in the participating countries. If successful, the TEC-MED model could inform future policies on aging care in culturally diverse regions, potentially influencing both national health strategies and EU-wide directives on elderly care.

## Data Accessibility Statement

The datasets used and/or analyzed during the current study are available from the corresponding author upon reasonable request.

## Figures and Tables

**Table:** 

AROPE	At risk of poverty or social exclusion
CG	Control group
IG	Intervention group
MD	Material deprivation
NIC	Nursing Interventions Classification
NOC	Nursing Outcomes Classification
RUD	Resource Utilization in Dementia
WHO	World Health Organization
